# Selective decontamination of the reactive air pollutant nitrous acid *via* node-linker cooperativity in a metal–organic framework†
†Electronic supplementary information (ESI) available. See DOI: 10.1039/c9sc01357a


**DOI:** 10.1039/c9sc01357a

**Published:** 2019-04-29

**Authors:** Devon T. McGrath, Michaela D. Ryan, John J. MacInnis, Trevor C. VandenBoer, Cora J. Young, Michael J. Katz

**Affiliations:** a Department of Chemistry , Memorial University of Newfoundland , St. John's , NL , Canada . Email: mkatz@mun.ca; b Department of Earth Sciences , Memorial University of Newfoundland , St. John's , NL , Canada; c Department of Chemistry , York University , Toronto , ON , Canada

## Abstract

Nitrous acid (HONO) is a reservoir of NO_*x*_ and an emerging pollutant having direct impacts on air quality, both in- and outdoors, as well as on human health. In this work, the amine-functionalized metal–organic framework (MOF), UiO-66-NH_2_, was investigated due to its potential to selectively decontaminate nitrous acid at environmentally relevant concentrations. UiO-66-NH_2_ proved to be effective in the removal of nitrous acid from a continuous gaseous stream. This is observed *via* the formation of an aryl diazonium salt that subsequently converts to a phenol with a concomitant release of nitrogen gas. This process is preceded *via* the formation of the nitrosonium cation (likely protonation from an acidic proton on the node). Thus, UiO-66-NH_2_ is capable of selectively converting the pollutant nitrous acid to benign products.

## Abstract 2

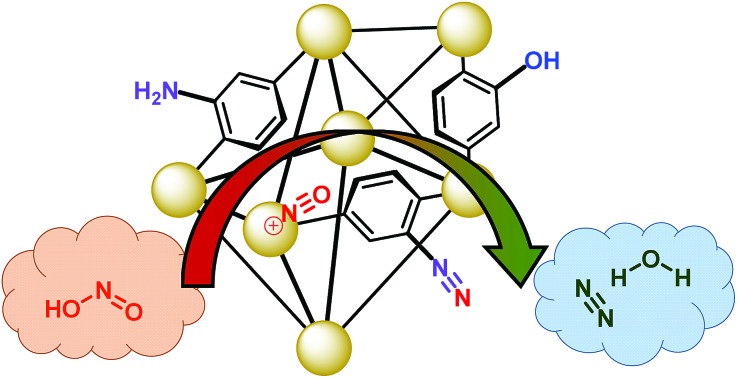
The environmental pollutant nitrous acid is rapidly and selectively sorbed and converted to benign products in the metal–organic framework UiO-66-NH_2_.

## 1 Introduction

Nitrogen dioxide (NO_2_) and nitric oxide (NO) are generated primarily from combustion processes in the transportation and energy sectors and are present in both indoor and outdoor air.^1^ Given the fact that nitrogen dioxide and nitric oxide are able to quickly interchange in the daytime atmosphere, they are often summed together as NO_*x*_.^2^ Environmentally, NO_*x*_ catalyze the deterioration of air quality *via* the formation of photochemical smog.^3^ The resultant poor air quality can negatively impact visibility, vegetation, and human health.^4^–^6^ The presence of nitrogen dioxide in the atmosphere has a direct negative impact on both the environment (*e.g.*, pollution)^7^,^8^ and human health (*e.g.*, cardiovascular and respiratory conditions).^4^,^9^–^11^ Indirectly, nitrogen dioxide can react further to form secondary pollutants that can be equally, if not more, dangerous to human health and the environment.^12^ One of the well-known secondary pollutants produced by nitrogen dioxide is nitrous acid (HONO).

Nitrous acid is a gas-phase pollutant that is emitted directly and formed indoors and outdoors *via* the reaction of nitrogen dioxide with surface-bound water (R1) and *via* the reaction of nitric oxide and hydroxyl radicals ((R2), reverse).^13^,^14^ Additionally, despite being advertised for the decontamination of nitrogen dioxide, titanium dioxide (TiO_2_), a common additive in building surfaces, windows, sidewalks, and paints,^15^,^16^ is implicated in the photochemical formation of nitrous acid.^12^ Nitrous acid subsequently photodissociates (R2) to form nitric oxide. In these regards, nitrous acid is a reservoir for NO_*x*_ that may be released at a later time, unless properly mitigated.^17^,^18^

R1 2NO_2_ + H_2_O (surface) → HONO + HNO_3_

R2

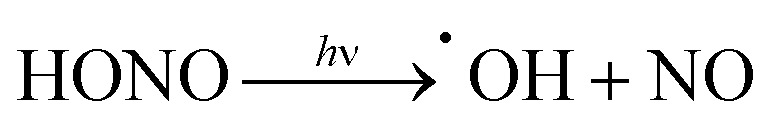




Nitrous acid has direct negative impacts on air quality.^12^,^19^ As a dominant radical source at the surface of the Earth, photolysis of nitrous acid (*via* indoor lighting or solar irradiation) releases the highly reactive hydroxyl radical (R2). These radicals are able to react directly with tissues in the respiratory tract or take part in atmospheric oxidation to produce other dangerous pollutants such as ground-level ozone and particulate matter smaller than 2.5 μm (PM_2.5_).^12^,^19^

Indoor concentrations of nitrous acid can be over 10 times higher than those present outdoors.^20^–^23^ Although nitrous acid is harmful to human health on its own,^15^,^24^ it will also react with directly-emitted gaseous amines (R3) or those found in cigarette or cooking smoke, resulting in the formation of carcinogenic nitrosamines, a component of third hand smoke.^25^–^28^

R3 R_2_NH + HONO → R_2_N–N=O + H_2_O

It is important to find an efficient method by which nitrous acid can be rapidly sequestered due to its highly reactive nature, the associated health risks, as well as its contribution to poor air quality. Given that nitrous acid is derived from, and a reservoir for, NO_*x*_, the deactivation of nitrous acid could be a more advantageous method to reduce NO_*x*_ emissions compared to direct sorption processes.

To address these challenges, we have turned our attention to the reactivity of nitrous acid within metal–organic frameworks (MOFs). MOFs are a class of porous materials composed of metal centers (nodes) and bridging organic ligands (linkers).^29^–^33^ The presence of different node geometries, as well as the synthetic tunability of linkers, *via* traditional organic chemistry routes, affords a high degree of tunability of the pore size, aperture, and reactivity.^34^ To that end, researchers have explored the utility of MOFs in applications such as chemical sensing, gas storage, chemical separations, and solar energy generation.^35^–^40^ Beyond these applications, specific MOFs have been featured in environmental decontamination strategies.^41^–^48^

**Fig. 1:**
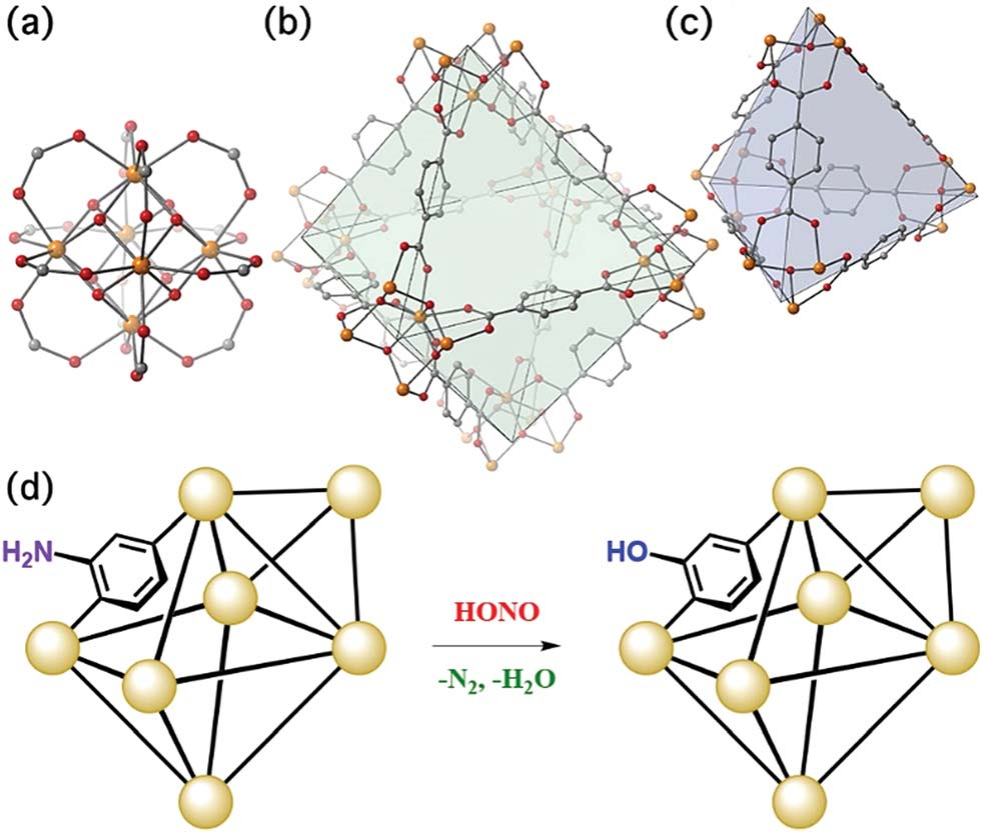
(a) The Zr_6_O_4_(OH)_4_^12+^ node of UiO-66 coordinated to 12 carboxylate anions. (b) The octahedral pore of UiO-66. (c) The tetrahedral pore of UiO-66. (d) Schematic representation of UiO-66-NH_2_ and the proposed reaction. For (a), (b), and (c): Zr – orange, C – gray, O – red.

The present work illustrates a chemisorption solution (Fig. 1) for nitrous acid (and ultimately NO_*x*_) where capacity, reactivity, and selectivity are greater than under physisorption conditions. In strongly-acidic aqueous solutions, it is known that the nitrite anion (NO_2_^–^) readily converts to nitrous acid and subsequently to the nitrosonium cation (NO^+^). The nitrosonium cation further reacts with aryl amines to form a diazonium salt, which in solution can be quenched to form aryl-halides, cyanides, and hydroxides.^49^,^50^ Herein, we demonstrate for the first time that similar chemistry is possible under heterogeneous conditions between nitrous acid gas and an appropriate MOF substrate, UiO-66-NH_2_ (Fig. 1).^51^–^54^ The Zr_6_O_4_(OH)_4_^12+^ cluster node of the UiO family (Fig. 1a) contains acidic protons (p*K*_a1_ = 3.5)^55^–^57^ on the μ_3_-bridging –OH groups. We hypothesize that these acidic groups are able to produce the nitrosonium cation inside the pores. The 2-aminoterephthalate linker (BDC-NH_2_) of UiO-66-NH_2_ contains the requisite aryl amine unit to react with the nitrosonium cation. Under humid conditions, the aryl diazonium is able to produce an aryl hydroxide, releasing nitrogen gas (Fig. 1d). Thus, UiO-66-NH_2_ is uniquely able to rapidly and selectively (*i.e.*, only nitrous acid is expected to undergo this chemistry) decontaminate gas-phase nitrous acid at environmentally-relevant concentrations (Fig. 1d).

## 2 Results and discussion

The sorption and desorption kinetics of nitrous acid were analyzed using a custom-built breakthrough experiment system (Fig. S2 and Section S3 in the ESI†). Briefly, nitrous acid was generated *in situ* by flowing dilute HCl gas at 50% relative humidity (RH) over a bed of solid sodium nitrite. The acid-displacement reaction produces gas-phase nitrous acid and solid sodium chloride; controlling concentration of the HCl and temperature results in a tunable nitrous acid source.^58^,^59^ For the purpose of these experiments, nitrous acid concentrations were maintained between 74 and 179 pg cm^–3^ (equivalent to 35 and 85 parts per billion by volume (ppbv)) at 50% RH; this is on the order of indoor and outdoor environmental concentrations.^20^,^60^ Approximately 10 mg of MOF was packed into a 1/4′′ perfluoroalkoxy (PFA) tube. Nitrous acid was flowed through the MOF and the downstream (post-MOF) concentrations were measured as a function of time on an American Ecotech oxides of nitrogen analyzer.

**Fig. 2:**
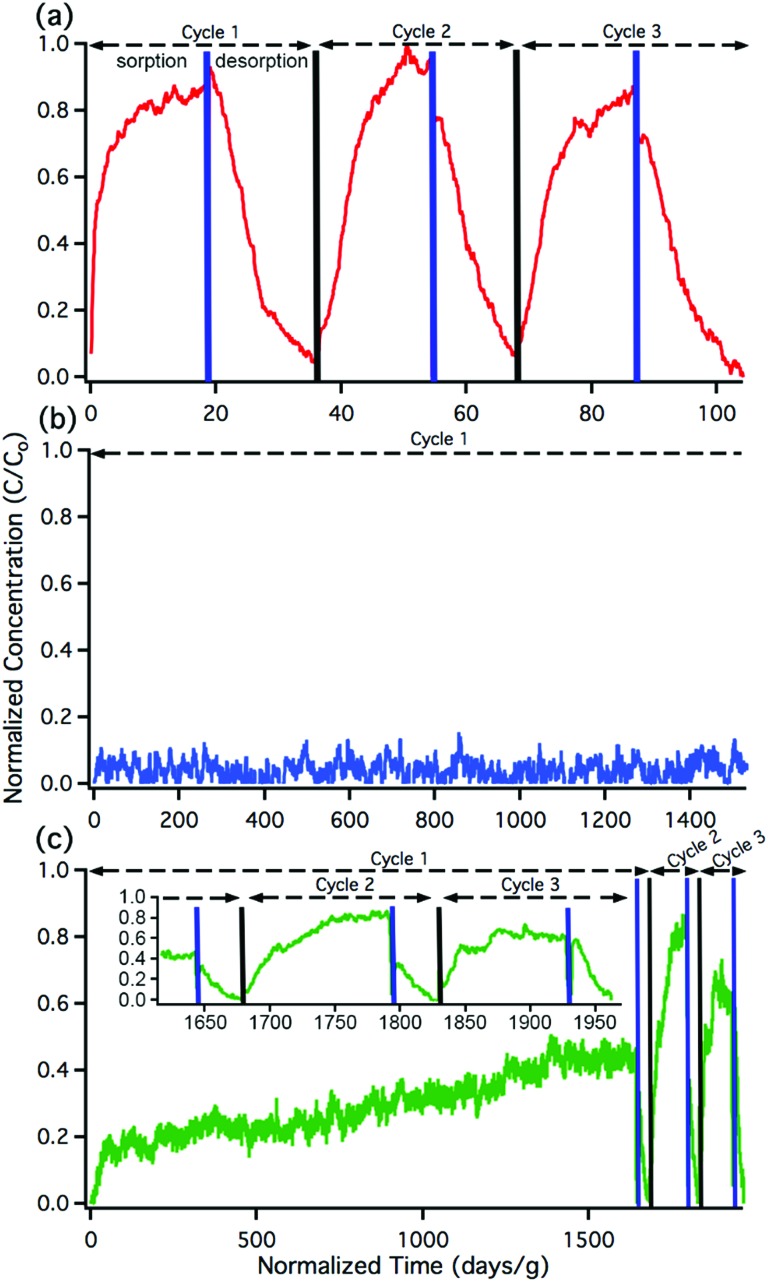
Nitrous acid breakthrough curves for (a) UiO-66, (b) UiO-66-NH_2_, and (c) UiO-66-(NH_2_)_1/6_; the inset illustrates an expanded view of cycles 2 and 3. The *x*-axis units are the same for all the plots. Note the difference in *x*-axis scale between (a), (b), and (c).

Breakthrough exposure experiments were initially conducted on UiO-66. UiO-66 contains the acidic, Zr_6_O_4_(OH)_4_^12+^, node and the benzenedicarboxylate (BDC) linker. Fig. 2a illustrates the downstream nitrous acid concentration as a function of time over three sorption and desorption cycles (Fig. S3† contains replicate data). Initially after exposure of UiO-66 to nitrous acid, the downstream concentration drops to zero. However, over a short period of time, the concentration of nitrous acid, post-UiO-66, begins to increase. This indicates that UiO-66 is not efficient at sequestering nitrous acid; 1 gram of this MOF would take approximately 15 days for saturation under these conditions (*i.e.*, concentration and flow rate). This is equivalent to 0.44% of the acidic protons or 1.8% of the nodes reacting with HONO (Table S2† compares various breakthrough units).

Desorption performed under 50% RH released approximately equal amounts of nitrous acid as was sorbed (evident in the sorption/desorption traces). This would suggest physisorption. However, when desorption is performed at 0% RH (Fig. S4†), then only a nominal amount of nitrous acid is desorbed. This indicates that nitrous acid is being chemically trapped in the pores of the MOF and water vapour is necessary for the desorption process. This is proposed to be due to the formation of the nitrosonium cation and a concomitant loss of water (*vide infra*).

Nitrogen gas adsorption studies, performed at 77 K, before and after three cycles of nitrous acid exposure indicate the Brunauer–Emmett–Teller (BET) surface area (SA) decreased by 20% (1770 m^2^ g^–1^ decreased to 1416 m^2^ g^–1^ respectively; Fig. S11†). Furthermore, the powder X-ray diffractogram (PXRD; Fig. S12†) of UiO-66 post nitrous acid exposure does not show signs of peak broadening or crystallinity loss. Thus, UiO-66 has not degraded over the course of the experiment.

With the sorptive nature of UiO-66 identified, we turned our attention to the amine-functionalized MOF, UiO-66-NH_2_. This MOF contains the acidic node and the aryl-amine-containing BDC-NH_2_ linker that is expected to react with the nitrosonium cation. As shown in Fig. 2b, the lack of downstream nitrous acid clearly illustrates that UiO-66-NH_2_ is highly reactive. Even after 1400 days per g (equivalent to 17 mg of HONO per g of MOF, or 11% of the linkers and 16% of the hydroxides converted; Table S2†), UiO-66-NH_2_ does not show any measurable quantity of nitrous acid downstream.^61^ Attempts to desorb nitrous acid from UiO-66-NH_2_ were unsuccessful; no nitrous acid or NO_*x*_ is observed post-MOF. This indicates that UiO-66-NH_2_ is acting as a highly reactive scavenger for nitrous acid. This data is consistent with chemisorption behaviour.

To verify that the MOF is not being degraded, the PXRD (Fig. S12†), and BET SA was measured before/after exposure to nitrous acid; a 14% reduction in overall BET SA was observed (Fig. S11†) after 30 days with no change in crystallinity (Fig. S12†). The lack of any notable change demonstrates that the MOF is stable under the experimental conditions. Even under high concentrations (>1 ppmv) of nitrous acid (Fig. S11†), only a 54% loss in BET surface area (1655 m^2^ g^–1^ decreased to 765 m^2^ g^–1^) is observed after a week-long exposure.

Replicate nitrous acid exposure cycles for UiO-66-NH_2_ could not be conducted because even with a small amount of MOF (10 mg), a full cycle for UiO-66-NH_2_ is expected to take upwards of 100 days. This issue was addressed by testing the sorption abilities of mixed linker MOF, UiO-66-(NH_2_)_1/6_ that contains 5/6 BDC and 1/6 BDC-NH_2_ linkers. By reducing the –NH_2_ content, the experimental time was reduced. As shown in Fig. 2c, the first sorption cycle shows a slow increase in downstream nitrous acid concentration as a function of time. This is inconsistent with the sorption data observed for UiO-66 but is consistent with the chemisorption data shown for UiO-66-NH_2_. In comparison, this breakthrough time is equivalent to 35 mg of HONO per gram of MOF and approximately 21% of the linkers and 31% of the acidic protons reacting (Table S2†). The difference between the first cycle of UiO-66-(NH_2_)_1/6_ and UiO-66-NH_2_ is due to the change in rate (*i.e.*, a decrease in contact time) associated with a six-fold decrease in BDC-NH_2_. The first desorption cycle for UiO-66-(NH_2_)_1/6_ shows that the amount of gas sorbed (the area above the sorption curve) is much larger than the amount of gas desorbed (the area below the desorption curve). This observation is consistent with chemisorption. Beyond the first cycle, the second and third cycles (Fig. 2c) illustrate data that is consistent with the sorption behaviour of UiO-66. We propose that during the first nitrous acid exposure cycle all (17%) of the BDC-NH_2_ sites are exhausted in UiO-66-(NH_2_)_1/6_. In cycles 2 and 3, the MOF is only capable of sorption behavior akin to that observed for UiO-66 (*i.e.*, the formation of the nitrosonium cation; *vide infra*). Similarly to both UiO-66 and UiO-66-NH_2_, UiO-66-(NH_2_)_1/6_ exhibits stable behaviour under reaction conditions with only a 15% decrease in BET SA before/after exposure experiments (1568 m^2^ g^–1^ decreased to 1338 m^2^ g^–1^ respectively; Fig. S11†).

To further elucidate the mechanism, solution phase NMR and solid-state IR spectroscopy were examined. However, under environmentally-relevant concentrations, the spectroscopy of UiO-66-NH_2_ was speculative. To address this, the nitrous acid concentration was increased *ca.* ten-fold; the breakthrough curve of UiO-66-NH_2_ resembled that of UiO-66-(NH_2_)_1/6_ indicating that at these concentrations the reaction kinetics are slower than the contact time.

**Fig. 3:**
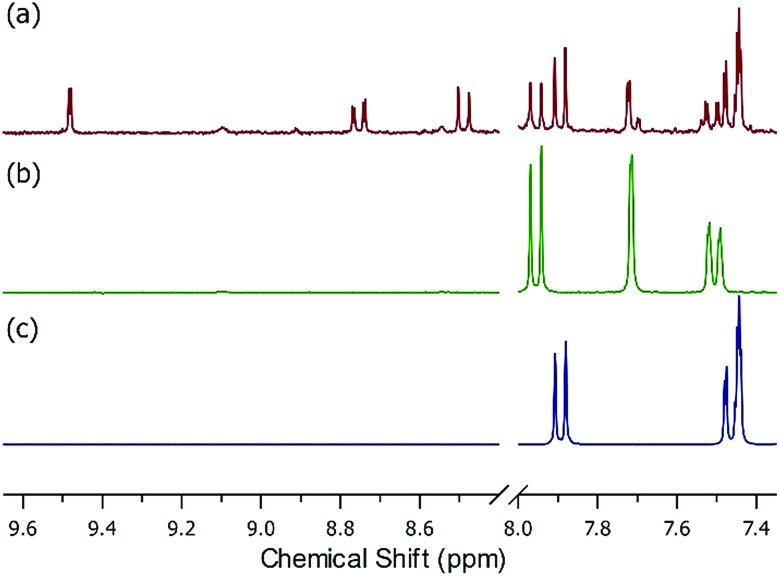
Solution phase NMR measured in DMSO-*d*_6_ (lock solvent) with 2 drops of D_2_SO_4_ of (a) UiO-66-NH_2_ post-nitrous acid exposure, (b) UiO-66-NH_2_, and (c) BDC-OH. Note the presence of strongly deshielded protons between 8.4 and 9.6 ppm (21% by NMR) as well as the presence of resonance consistent with BDC-OH (45% by NMR) and the parent BDC-NH_2_ (34% by NMR) in (a). Each component integrated to a 1 : 1 : 1 ratio as expected.

Starting with UiO-66, at moderate (650 ppbv mixing ratio) nitrous acid concentrations (Fig. S8†), the NMR showed no change before/after nitrous acid exposure. The NMR of UiO-66-NH_2_ post-nitrous acid exposure (Fig. 3a) shows that three different mono-substituted BDC linkers are now present in solution. Considering the sorption profile of UiO-66-NH_2_ (Fig. 2b) did not begin to show nitrous acid breakthrough, remnant BDC-NH_2_ (Fig. 3b *vs.* a) is expectedly present (34% by NMR). Most interesting is the presence of a strongly deshielded BDC-*X* from 8.4 ppm to 9.6 ppm (21% by NMR). We propose that this is BDC-N_2_^+^, the electron withdrawing (*i.e.*, deshielding) product of the rapid reaction between nitrosonium and an aryl amine.^42^ The NMR (Fig. 3) illustrates that once the node reacts with nitrous acid to form the nitrosonium cation, then BDC-NH_2_ rapidly reacts with the cation to form BDC-N_2_^+^ (Fig. 5). Given that diazonium salts are inherently unstable, and a 50% RH gas stream is used in the experiments (Fig. 2), the third moiety in the NMR is BDC-OH (Fig. 3a *vs.* c; 45% by NMR). At even higher (>1 ppmv) nitrous acid concentration (Fig. S7†),^62^ NMR analysis of the MOF post-nitrous acid exposure only shows signs of the formation of BDC-N_2_^+^. This demonstrates that BDC-OH forms as a result of the formation of BDC-N_2_^+^.

The presence of BDC-N_2_^+^ is further corroborated by IR spectroscopy. Unlike pristine UiO-66-NH_2_, after exposure to nitrous acid, a new vibration at 2283 cm^–1^ is observed (Fig. S10†). This vibration is consistent with the stretching frequency of a diazonium; this does not appear in the spectra for nitrous acid-exposed UiO-66 (Fig. S10†).^62^,^63^

**Fig. 4:**
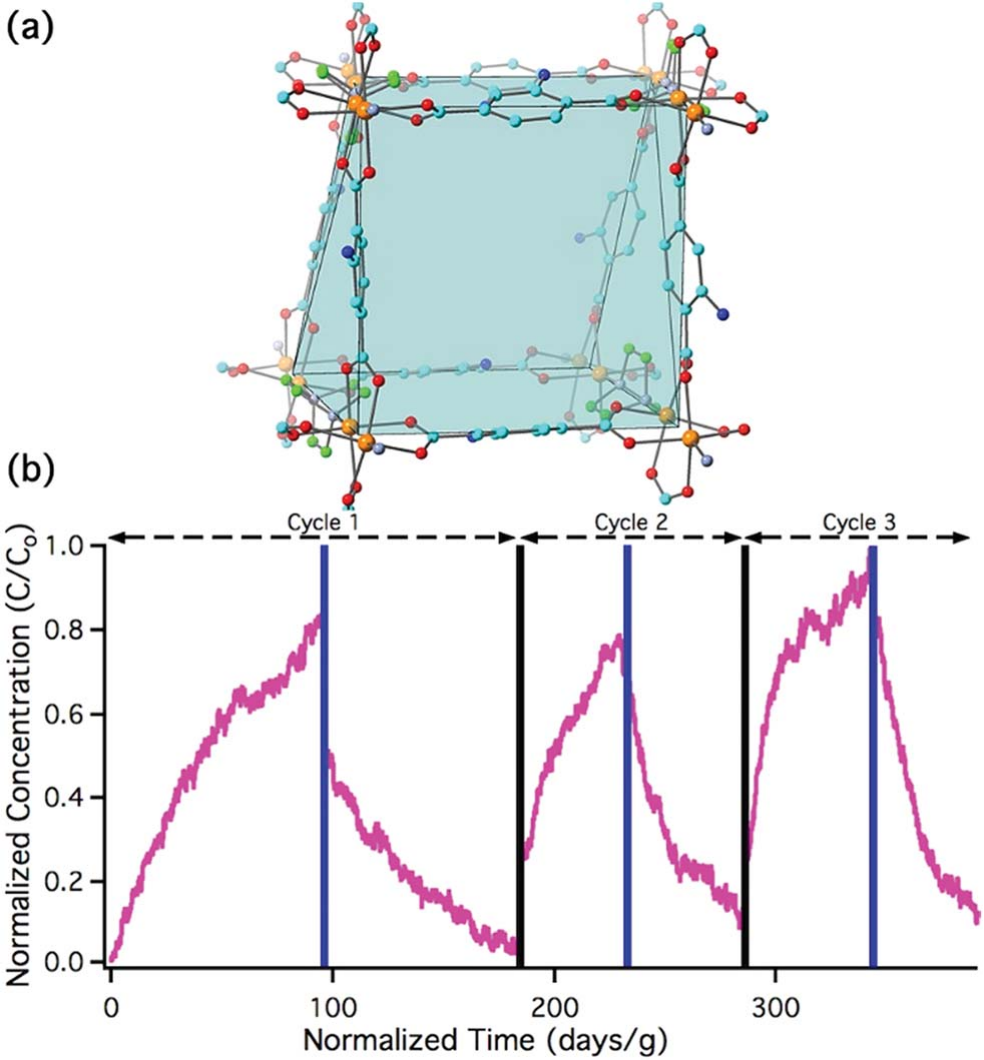
(a) Crystal structure of Zn_2_(BDC-NH_2_)_2_(DABCO) showing how 2D paddlewheel sheets of Zn_2_(BDC-NH_2_)_2_ are pillared *via* DABCO units: Zn – orange, C – cyan/green for BDC/DABCO, N – light blue/blue for BDC/DABCO, O – red. (b) Nitrous acid breakthrough curves for three adsorption/desorption cycles of Zn_2_(BDC-NH_2_)_2_(DABCO); these breakthrough experiments were run between 100 and 160 ppbv nitrous acid mixing ratios to ensure that the MOF has optimal interaction time with nitrous acid. Stoichiometric equivalents for the first breakthrough can be found in Table S2.†

Breakthrough experiments were repeated with the pillared paddlewheel MOF, Zn_2_(BDC-NH_2_)_2_(DABCO) (Fig. 4a, where DABCO is 1,4-diazobicyclo[2.2.2]octane).^29^ This MOF contains the BDC-NH_2_ reactive site without the acidic node of UiO-66-NH_2_. As shown in Fig. 4b, the downstream concentration of nitrous acid as a function of time is consistent with physisorption behaviour rather than the chemisorption behaviour of UiO-66-NH_2_ (Fig. 2b *vs.*4b).^64^ NMR analysis of dissolved Zn_2_(BDC-NH_2_)_2_(DABCO) shows the presence of BDC-NH_2_ and DABCO with no notable BDC-N_2_^+^ or BDC-OH (Fig. S9†). The BDC-NH_2_ moiety is non-reactive to nitrous acid in the absence of the Zr_6_O_4_(OH)_4_^12+^ node. This confirms the importance of the UiO-66 node to the heterogenous reaction.

**Fig. 5:**
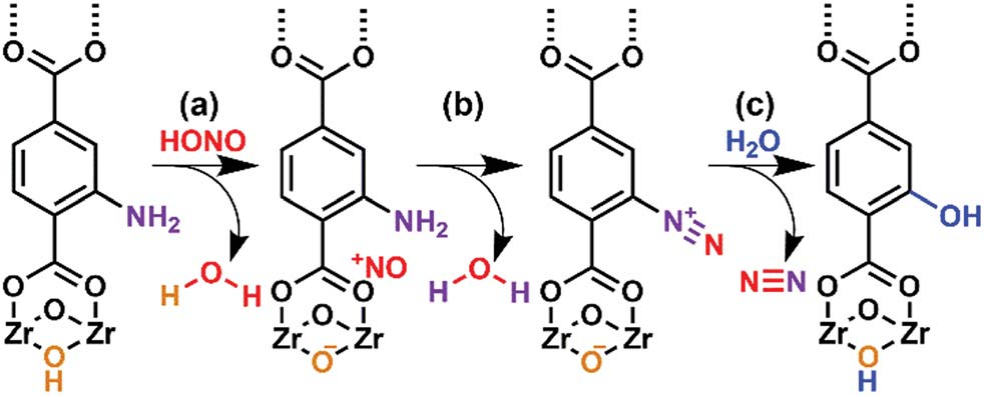
Hypothesized mode of action for the reaction between nitrous acid and UiO-66-NH_2_. (a) The reaction of gas-phase nitrous acid with –OH group on Zr-based node producing the nitrosonium cation and water. (b) The formation of the diazonium intermediate with concomitant loss of water. (c) The diazonium intermediate reacting with water producing UiO-66-OH with nitrogen gas as a benign reaction byproduct.

As illustrated in Fig. 5, UiO-66-NH_2_ operates in a cooperative method. We propose that the node is responsible for producing pore-bound nitrosonium cations (with the node acting as the counterion), *via* loss of water. The nitrosonium cation subsequently reacts with BDC-NH_2_ to form UiO-66-(N_2_^+^)^–^, once again releasing water. The nitrosonium-containing MOF further reacts with the gaseous water to produce UiO-66-OH.

## 3 Conclusions

In conclusion, we have demonstrated that UiO-66-NH_2_ is a rapid and selective scrubber of nitrous acid. Although UiO-based MOFs have been shown by others to excel at nitrogen dioxide sequestration,^42^,^48^ the mechanisms of interaction of UiO-66-NH_2_ with nitrogen dioxide and nitrous acid are different (*i.e.*, physisorption *vs.* chemisorption). The reactivity and selectivity of UiO-66-NH_2_ to nitrous acid should not be affected by the presence of nitrogen dioxide. The products of this newly-demonstrated heterogeneous reaction are nitrogen gas and water. Considering that nitrous acid is a reservoir for NO_*x*_, then scrubbing nitrous acid acts as a denoxification method. That is, it returns inert nitrogen gas as a product instead of cycling oxidized species. The denoxification reaction performed by this material is unusual in environmental remediation. Unlike a vehicle catalytic converter, this material can be effectively applied post-emission at dilute concentrations and low temperatures.

Considering the substantial amounts of nitrous acid found indoors, UiO-66-NH_2_ can be utilized in removal of nitrous acid from homes, particularly those where combustion devices such as gas stoves, fireplaces, and candles are used. By implementing MOF filters in oven range hoods and air exchange systems there is the potential for the removal of indoor nitrous acid in homes and businesses.

Furthermore, given that the chemistry presented in this work is selective to nitrous acid, UiO-66-NH_2_ is a promising candidate for environmental monitoring of nitrous acid in real world scenarios.^20^,^65^ Present techniques, albeit very well established, are cumbersome. UiO-66-NH_2_ can be used to get a better idea of sources and sinks of nitrous acid in the environment.

## Conflicts of interest

There are no conflicts to declare.

## Supplementary Material

Supplementary information
