# Supplementary material for: Selective decontamination of the reactive air pollutant nitrous acid via node-linker cooperativity in a metal–organic framework
Source: Chem Sci. 2019 Apr 29;10(21):5576–81. doi: 10.1039/c9sc01357a (PMC6553375; doi:10.1039/c9sc01357a)
Supplement: Supplementary file 1 [file SC-010-C9SC01357A-s001.pdf]

Electronic Supplementary Material (ESI) for Chemical Science.
This journal is © The Royal Society of Chemistry 2019

[Electronic Supporting Information (ESI) to accompany:]

Selective Decontamination of the Reactive Air Pollutant Nitrous Acid via Node-Linker Cooperativity in a Metal-Organic Framework

Devon T. McGrath,[a] Michaela D. Ryan,[a] John J. MacInnis,[a] Trevor C. VandenBoer,[b,c] Cora J. Young,[a,c] Michael J. Katz[a,*]

[a] *Department of Chemistry, Memorial University of Newfoundland, St. John's, NL, Canada.*

[b] *Department of Earth Sciences, Memorial University of Newfoundland, St. John's, NL, Canada.*

[c] *Present address: Department of Chemistry, York University, Toronto, ON, Canada.*

* *mkatz@mun.ca*

Table of Contents

S1 Synthesis S3
S1.1 Synthesis of 2-hydroxyterephthalic acid S3
S1.2 Synthesis of UiO-66 and UiO-66-NH_2 ..... S4
S1.3 Synthesis of $Zn_2(BDC\text{-}NH_2)_2(DABCO)$ S5
S2 Characterization S5
S2.1 Nuclear Magnetic Resonance (NMR) S5
S2.2 Infrared (IR) Spectroscopy S5
S2.3 Surface Area (SA) Analysis S6
S2.4 Powder X-Ray Diffraction (PXRD) S6
S3 Nitrous Acid Measurements S7
S3.1 Custom Instrumentation for Nitrous Acid Generation S7
S3.2 Quantification of Nitrous Acid in MOF Breakthrough Experiments S8
S3.3 Oxides of Nitrogen Analyzer S9
S4 MOF Characterization S10
S4.1 Triplicate Nitrous Acid Breakthroughs of UiO-66 ..... S11
S4.2 Nitrous Acid Breakthroughs of UiO-66 with 0% RH Desorption S12
S4.3 Triplicate Nitrous Acid Breakthroughs of UiO-66-$(NH_2)_{1/6}$ S13
S4.4 Triplicate Nitrous Acid Breakthroughs of $Zn_2(BDC\text{-}NH_2)_2(DABCO)$ S14
S4.5 NMR Data for Pre/Post-Nitrous Acid Exposure S15
S4.6 IR Data for Pre/Post-Nitrous Acid Exposure S18

S4.7 Gas Adsorption Data for Pre/Post-Nitrous Acid Exposure S19
S4.8 PXRD Data Pre/Post-Nitrous Acid Exposure S20
S5 References S20

S1 Synthesis

Unless otherwise noted, all reagents were purchased from commercial sources and used without further purification.

Table S1: List of reagents, and their suppliers, used in this work.

Reagent	Supplier	Reagent	Supplier
1,4-diazabicyclo[2.2.2]octane (DABCO)	Sigma-Aldrich	2-aminoterepthalic acid ($H_2BDC-NH_2$)	Sigma-Aldrich
2-bromoterephthalic acid ($H_2BDC-Br$)	Sigma-Aldrich	Copper (Cu) powder	Fisher Scientific Company
Dimethylsulfoxide-*d*$_6$ (DMSO- *d*$_6$)	Cambridge Isotope Laboratories, Inc.	Hydrochloric acid (HCl)	Caledon Laboratory Chemicals
Methanol (MeOH)	ACP Chemicals	*N,N*-dimethylformamide (DMF)	Fisher Chemical
Sodium acetate (NaOAC)	Sigma-Aldrich	Sodium hydroxide (NaOH)	ACP Chemicals
Sodium nitrite ($NaNO_2$)	Sigma-Aldrich	Sulfuric acid-*d*$_2$ (D_2SO_4)	Sigma-Aldrich
Terephthalic acid (H_2BDC)	Sigma-Aldrich	Zirconium tetrachloride ($ZrCl_4$)[a]	Sigma-Aldrich
Zinc nitrate hexahydrate ($Zn(NO_3)_2{\cdot}6H_2O$)	Sigma-Aldrich		

[a] was stored under nitrogen gas prior to use.

S1.1 Synthesis of 2-hydroxyterephthalic acid

The ligand 2-hydroxyterephthalic acid ($H_2BDC-OH$) was synthesized according to the literature preparation of Zhao *et al.*[1] A scaled down synthesis was carried out. 1.01 g (5.0 mmol) of $H_2BDC-Br$ and 0.59 g (15.0 mmol) of NaOH were mixed in 20 mL of water.

1.02 g (12.5 mmol) of NaOAc and 0.01 g (0.32 mmol) of Cu powder were added while stirring. The solution was refluxed for 30 hours before a few drops of 1 M NaOH were added to ensure Litmus paper remained blue. The solution was further heated for 6 hours. Subsequently, the terephthalic acid-based compounds were precipitated by addition of concentrated HCl; a white precipitate was filtered and dried. ^{1}H NMR (300 MHz, DMSO-d_6) δ 7.90 (dd, J = 8.1, 0.6 Hz, 1H), 7.47 (dd, J = 7.9, 1.6 Hz, 1H), 7.45 (dd, J = 1.6, 0.6 Hz, 1H).

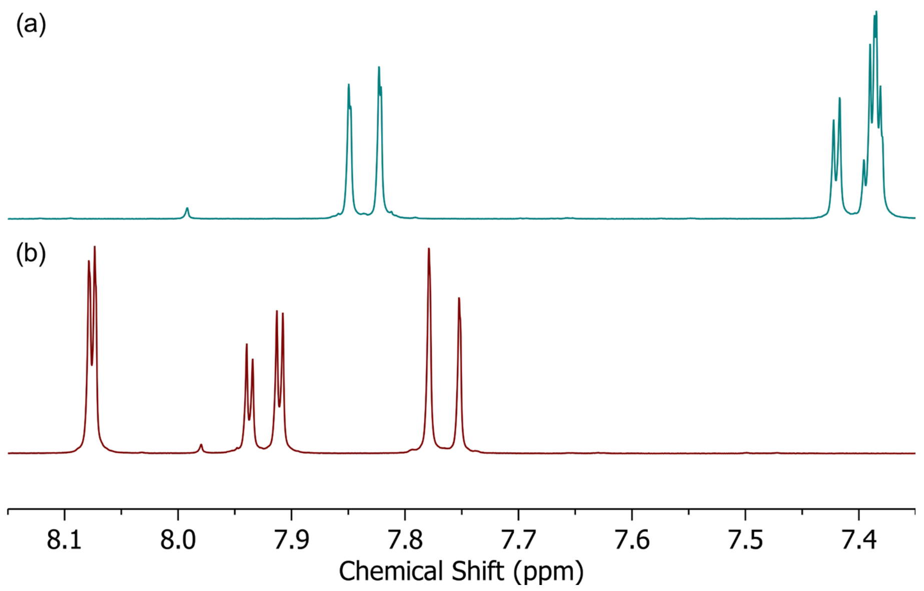

Fig. S1. NMR spectrum of synthesized (a) H_2BDC-OH and (b) the starting materials, H_2BDC-Br.

S1.2 Synthesis of UiO-66 and UiO-66-NH_2

UiO-66 and UiO-66-NH_2 were synthesized using the same procedure by Katz *et al.*[2] Each MOF was synthesized in 25 mL Duran® glass bottles. Using the preparation of Zwoliński *et al.*[3] the isolated UiO-66-NH_2 product was refluxed in 25 mL of MeOH for 24 h to remove any formylated UiO-66-NH_2. The purified UiO-66-NH_2 was filtered, dried, and stored wrapped in tinfoil in the dark.

S1.3 Synthesis of $Zn_2(BDC\text{-}NH_2)_2(DABCO)$

Synthesis of $Zn_2(BDC\text{-}NH_2)_2(DABCO)$ was carried out using the literature preparation from McGrath *et al.*[4] In a 25 mL Duran® glass bottle 0.0947 g (0.844 mmol) DABCO was sonicated to dissolution in 7 mL DMF. To this, 0.5010 g (1.684 mmol) of $Zn(NO_3)_2{\cdot}6H_2O$ was added along with another 7 mL aliquot of DMF and the resultant solution was sonicated to dissolution. Finally, 0.2675 g (1.526 mmol) of $H_2BDC\text{-}NH_2$ was added with an additional 7 mL aliquot of DMF and the final solution was sonicated to dissolution. Subsequently, the sample was placed into a 120 °C oven for 48 h. After 48 h, the sample was removed from the oven and the cooled crystals were transferred to a 50 mL centrifuge tube and centrifuged for 5 min at 5000 rpm. The mother liquor was then decanted off and the sample was washed with three 10 mL aliquots of DMF followed by three 10 mL aliquots of MeOH under the same centrifugation conditions mentioned above. The crystals were characterized by NMR.

S2 Characterization

S2.1 Nuclear Magnetic Resonance (NMR)

^{1}H-NMR analyses were measured using a Bruker AVANCE III 300 MHz NMR. The solution phase NMR of all MOF samples were prepared using ca. 5 mg of MOF first dissolved in 2-3 drops of D_2SO_4. Once dissolved, approximately 1 mL of DMSO-d_6 was added as the NMR lock solvent. It should be noted that small chemical shift differences can occur between samples due to the change in dielectric constant caused by the slight variance in pH between samples.

S2.2 Infrared (IR) Spectroscopy

A Bruker Alpha FTIR spectrometer equipped with an ATR crystal was used for IR characterizations. The scanning range was 500 cm^{-1} – 4000 cm^{-1}.

S2.3 Surface Area (SA) Analysis

All samples were thermally activated using a Micromeritics Smart VacPrep gas adsorption sample preparation instrument. The samples were initially heated to 90 °C while a vacuum level below 1.00 mmHg was reached at 5.00 mmHg s^{-1}. Subsequently, the samples were heated to 150 °C for 500 min at a ramp rate of 5 °C min^{-1}. The nitrogen-accessible gas adsorption isotherms (77 K) were measured using a 3Flex Surface Characterization instrument, with the accompanying MicroActive software suites. The accessible surface area was calculated based on Brunauer–Emmett–Teller (BET) theory using the 4-point criterion.[5]

S2.4 Powder X-Ray Diffraction (PXRD)

Powder X-ray diffractograms were measured on a Rigaku Ultima IV X-ray diffractometer with a Cu X-ray source operating at 40 kV x 44 mA (1.76 kW) and a scintillation counter detector. A continuous scan mode was applied between 2θ = 5° - 30° with a sampling width of 0.020° and a scan rate of 1.000 °/min.

S3 Nitrous Acid Measurements

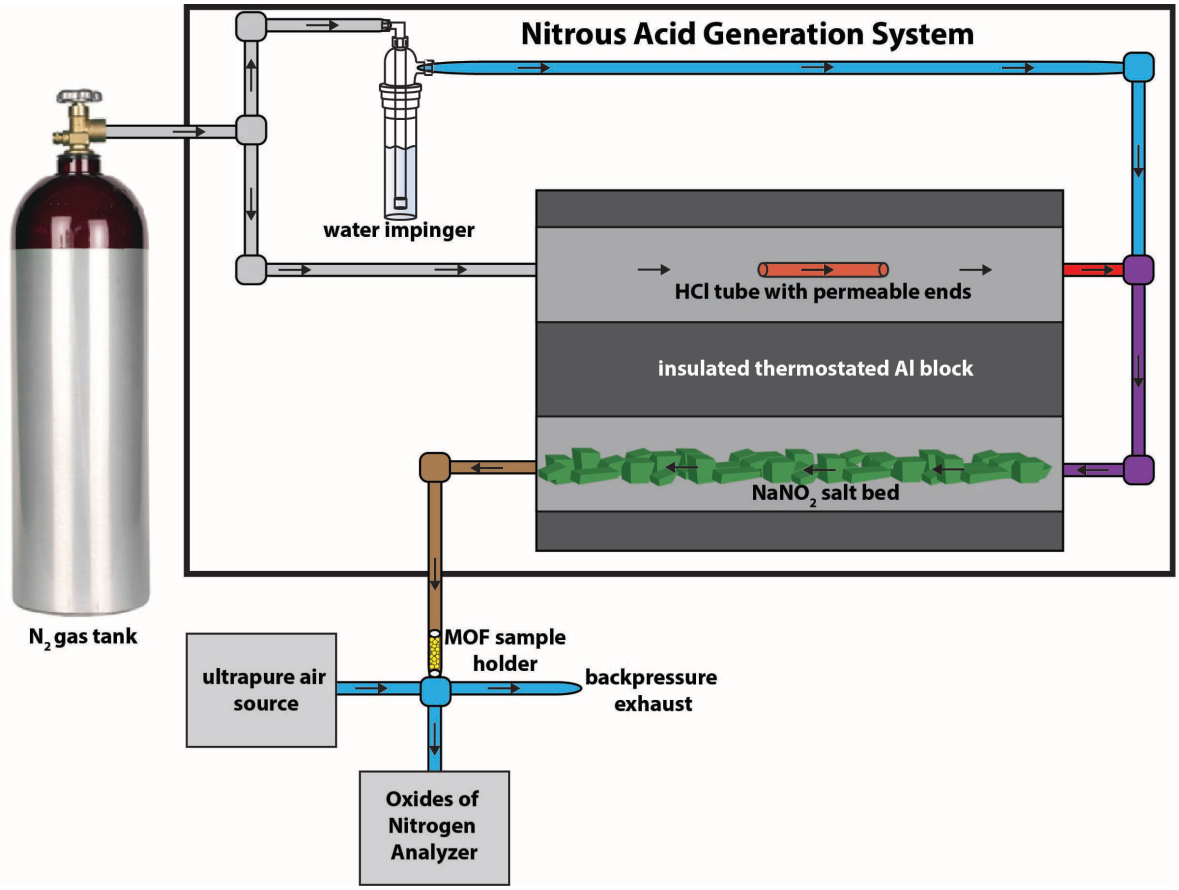

Fig. S2. Schematic of the nitrous acid gas-phase source connected to the MOF sample holder and the downstream air generator and detector.

S3.1 Custom Instrumentation for Nitrous Acid Generation

The generation of gas-phase nitrous acid was based on an acid-displacement reaction (Fig. S2).[6-7] To accomplish this, an HCl permeation tube is necessary. A 5 cm long PFA tube (ID = 3 mm, OD = 5 mm) is capped at one end with a 0.5 cm long 1/8" polytetrafluoroethylene (PTFE) rod. The tube is then filled with 2.5 M HCl and the open end is sealed with an identical PTFE rod. A heat gun is used to soften the PFA tube at both ends for insertion of the PTFE caps in order to seal the permeation tube. The PTFE material is porous to HCl gas.

To make a stable/tunable nitrous acid source, a 120 sccm stream of nitrogen gas is divided. Each 60 sccm stream flows through PFA tubing (ID = 1 mm, OD = 3 mm), with one stream

bubbled through water to produce a 100% relative humidity (RH) nitrogen stream. The other stream flows through a heated channel in an aluminum block containing the porous HCl permeation tube. The two streams are recombined to yield 50% RH gaseous HCl. The humid HCl flows over solid $NaNO_2$ residing in the second channel of the heating block to produce gas-phase nitrous acid. This nitrous acid gas is subsequently used for MOF breakthrough experiments. Higher Al block temperatures result in higher mass-emission rates of HCl and increased nitrous acid production. The output concentration of the nitrous acid source is measured using an Ecotech Serinus 40 Oxides of Nitrogen Analyzer (American Ecotech, Warren, RI).

S3.2 Quantification of Nitrous Acid in MOF Breakthrough Experiments

To measure the time-resolved downstream nitrous acid concentration through various MOFs, a thermally activated MOF (activated under vacuum at 100 °C) sample (ca. 10 mg) was packed into a 4 cm PFA tube between two plugs of glass wool (ca. 13 mg each). Downstream nitrous acid measurements made through only glass wool (i.e., in the absence of MOF) resulted in negligible nitrous acid uptake compared to that seen in the presence of MOF. The tubing containing the MOF was connected to the nitrous acid source via push-to-connect fittings. The downstream concentration of nitrous acid through the MOF was measured on the Serinus 40. Pre- and post-MOF gas flow was measured consistently during breakthrough experiments with no observed flow/pressure reduction.

Nitrous acid desorption experiments were done by replacing the nitrous acid source with a 50% RH nitrogen gas stream at 120 sccm. The downstream concentration was monitored with the Serinus 40.

S3.3 Oxides of Nitrogen Analyzer

To measure nitrous acid concentrations, the Serinus 40 was used. The instrument is designed to measure NO_x (NO_2 + NO) in the range of 0-20 ppm with a sensitivity of 0.4 ppb. The instrument operates at 0.1 Hz and averages to 1 min measurements. A minimum flow rate of 1500 sccm is needed for the instrument to operate correctly. To that end, a 1500 sccm flow of ultrapure air (GasCal 1100 + 8301 Compressed Air source from American Ecotech, Warren, RI) is combined with the experimental flow at the instrument inlet (downstream of the MOF) through a backpressure exhaust. This ensures nitrous acid concentrations are within the calibrated and linear response range of the instrument and in excess of its intake flow rate; the data accounts for the dilution factor from this.

The general detection principle of the Serinus 40 involves reacting nitric oxide with ozone (O_3) to generate excited state NO_2^*. During luminescent relaxation, a photon is released and detected by a photomultiplier.[8] Using this technique, nitric oxide can be measured easily in the O_3-reaction channel. Other nitrogen oxides must first be converted to nitric oxide before they can be measured. The mechanisms for nitrogen dioxide and nitrous acid analysis are synonymous and involve converting either species into nitric oxide using the Mo catalyst heated to 325 °C in the NO_x channel. Thus, nitrogen dioxide is determined by difference between the NO_x and nitric oxide channels. If only nitrous acid is introduced to the NOx channel, then the instrument can also be exploited to measure nitrous acid, which is well documented.[9] To ensure that we are producing nitrous acid and not nitrogen dioxide, a control experiment is run periodically in which a sodium carbonate (Na_2CO_3)-coated annular denuder (URG Corp., Chapel Hill, NC) is used to remove nitrous acid from our gas stream.[10-12] Under these conditions, the analyzer is only measuring nitrogen

dioxide and nitric oxide. With the denuder in line, no downstream nitrogen dioxide, or nitric oxide were observed. Thus, our nitrous acid source was producing nitrous acid and no NO_x.

The mass-emission rate of nitrous acid was determined by bubbling the output flow through 1 mM KOH solution. Under this condition, nitrous acid is converted back to the nitrite anion. The nitrite concentration was subsequently measured by anion ion chromatography with conductivity detection (IC). The separation was performed using a ThermoScientific ICS-2100 fitted with AS11-HC guard and analytical columns, a KOH eluent generator system (EGC-III), running a gradient elution program at 30 °C and a mobile phase flow of 1.5 mL/min, followed by suppressed conductivity detection.[13]

S4 MOF Characterization

Table S2: Breakthrough times for the first sorption cycle featured in the main text represented in different units (column 2, 3, and 4 correspond to the first sorption curve in Fig. 2a, 2b, and 2c respectively, while column 5 corresponds to the first sorption curve in Fig. 4b).

	UiO-66			
	BDC	$BDC\text{-}NH_2$	$BDC\text{-}(NH_2)_{1/6}$	$Zn_2(BDC\text{-}NH_2)_2(DABCO)$
**Exposure ppbv[a]**	75	35	70	105
**days/g**	20	1500	1500	100
**mg HONO/g MOF**	0.50	17	35	3.5
**mmole HONO/g MOF**	0.011	0.37	0.74	0.074
**mole HONO/mole MOF**	0.018 (0.016)[b]	0.65 (0.57)[b]	1.2 (1.1)[b]	0.045
**mole HONO/mole linker**	0.0029 (0.0039)[b]	0.11 (0.14)[b]	0.21[c] (0.28)[b]	0.022
**mole HONO/mole metal**	0.0029 (0.0026)[b]	0.11 (0.09)[b]	0.21[c] (0.18)[b]	0.022
**mole HONO/mole acid[d]**	0.0044 (0.0034)[b]	0.16 (0.12)[b]	0.31 (0.24)[b]	N/A

[a] Average value of nitrous acid produced prior to the start of breakthrough measurements (i.e., C_0).
[b] Values in brackets calculated based on 2 missing linkers and the addition of two charge compensating hydroxides and two coordination number compensating water units.
[c] Only 1 in 6 linkers in UiO-66-$(NH_2)_{1/6}$ are $BDC\text{-}NH_2$. The maximum uptake would equate to 0.17 moles HONO/moles linker (6 linkers per node); the measured value is within error of the expected value.
[d] The acidic proton comes from the $Zr_6O_4(OH)_4^{12+}$ node. The μ_3-OH units contain acidic protons.

S4.1 Triplicate Nitrous Acid Breakthroughs of UiO-66

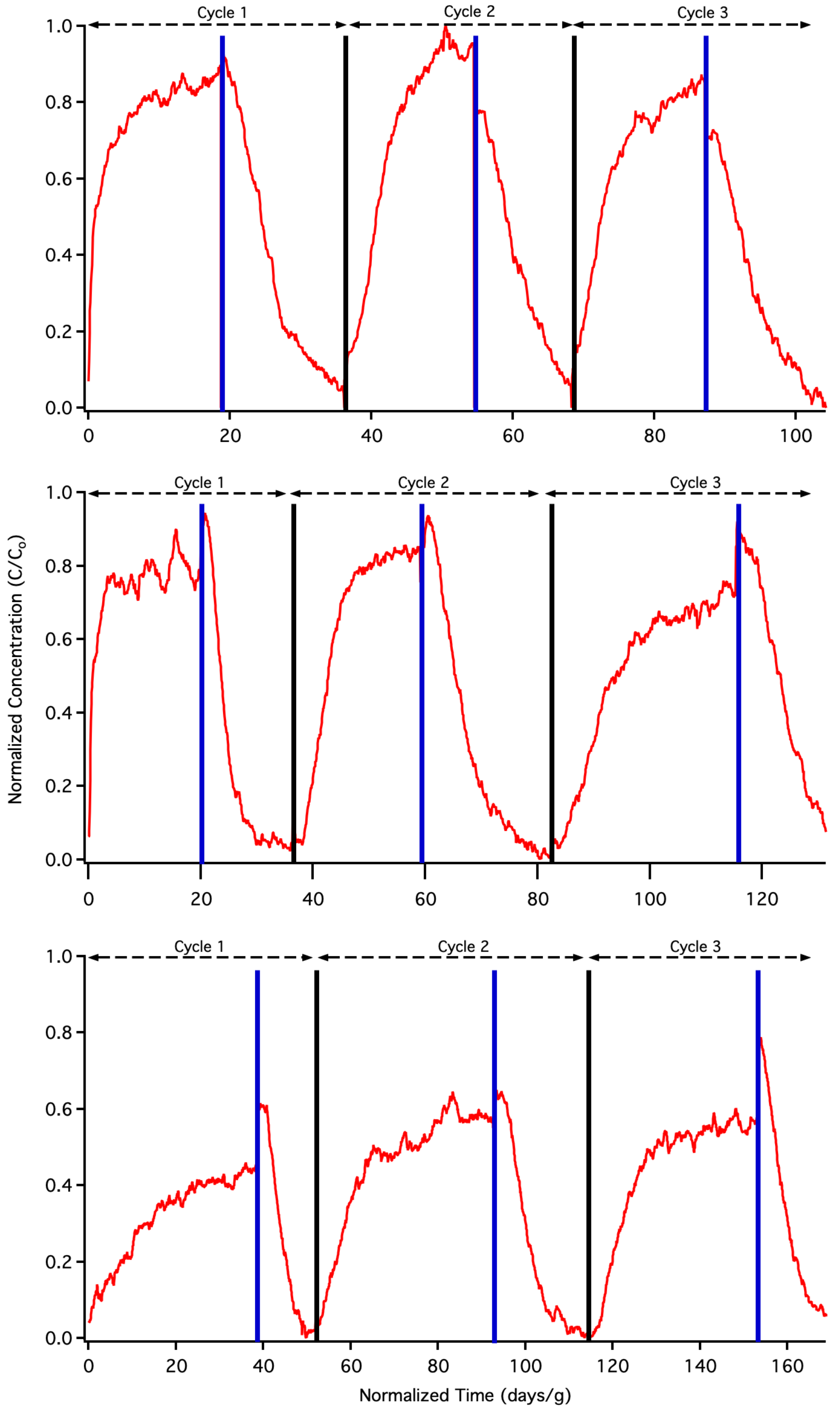

Fig. S3. Nitrous acid breakthrough curves for three samples of UiO-66. Each sample was run through 3 sorption and desorption cycles under 50% RH. Blue lines separate sorption and desorption curves, while black lines separate different cycles on the same MOF sample.

S4.2 Nitrous Acid Breakthroughs of UiO-66 with 0% RH Desorption

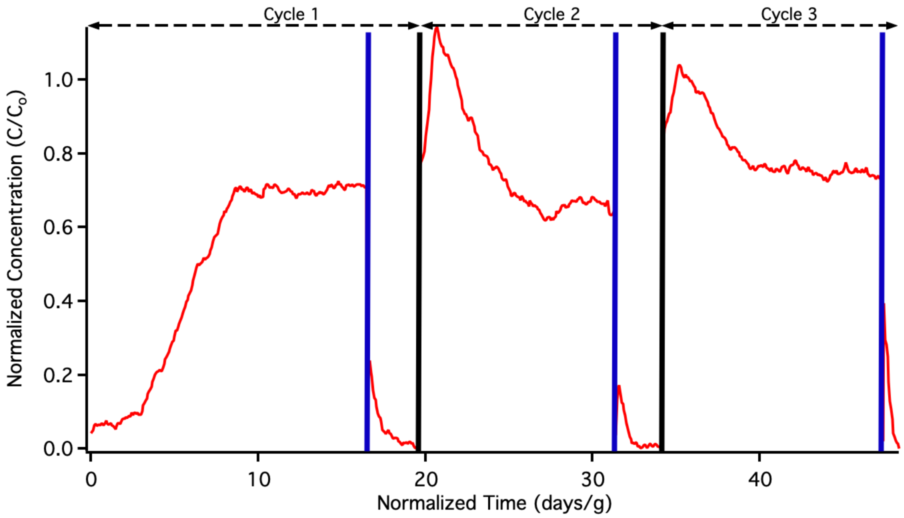

Fig. S4. Nitrous acid breakthrough curves for one sample of UiO-66. The sample was run through three sorption and desorption cycles. Each desorption cycle flowed a dry (0% RH) N_2 gas stream through the sample. Blue lines separate sorption and desorption curves, while black lines separate different cycles on the same MOF sample. Note the minimal desorption using 0% RH, and the spike in concentration when the MOF is exposed in subsequent (cycles 1 and 2) sorption cycles due to the presence of 50% RH in the subsequent HONO gas stream sorption cycle. The present work illustrates that, unlike the 50% RH desorption in Fig. 2 and Fig. S3, no nitrous acid is desorbed under 0% RH desorption gas. This demonstrates that nitrous acid is chemically trapped as the nitrosonium cation within UiO-66.

S4.3 Triplicate Nitrous Acid Breakthroughs of UiO-66-$(NH_2)_{1/6}$

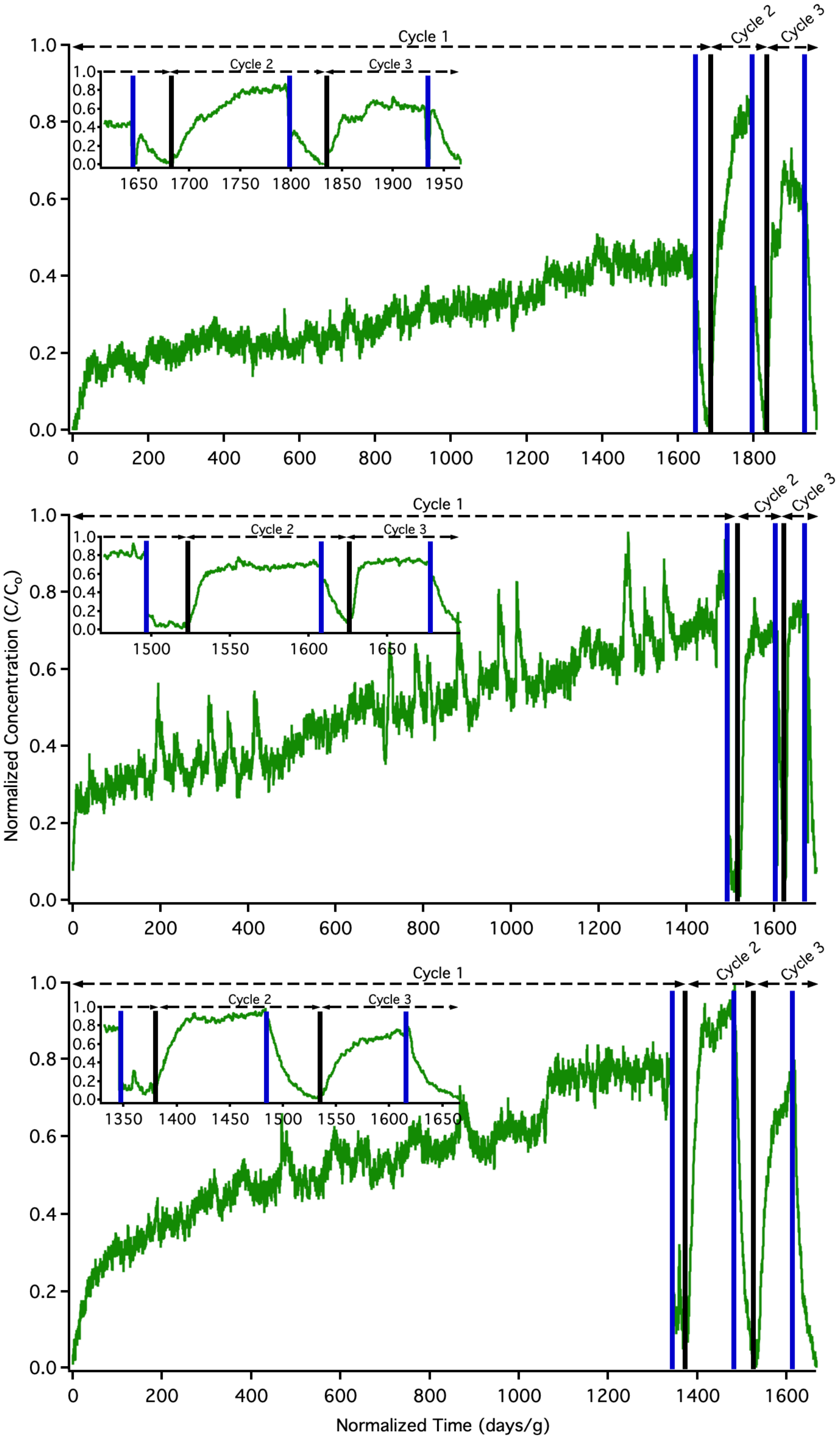

Fig. S5. Nitrous acid breakthrough curves for three samples of UiO-66-$(NH_2)_{1/6}$. Each sample was run through 3 sorption and desorption cycles under 50% RH. Blue lines separate sorption and desorption curves, while black lines separate different cycles on the same MOF sample.

S4.4 Triplicate Nitrous Acid Breakthroughs of $Zn_2(BDC\text{-}NH_2)_2(DABCO)$

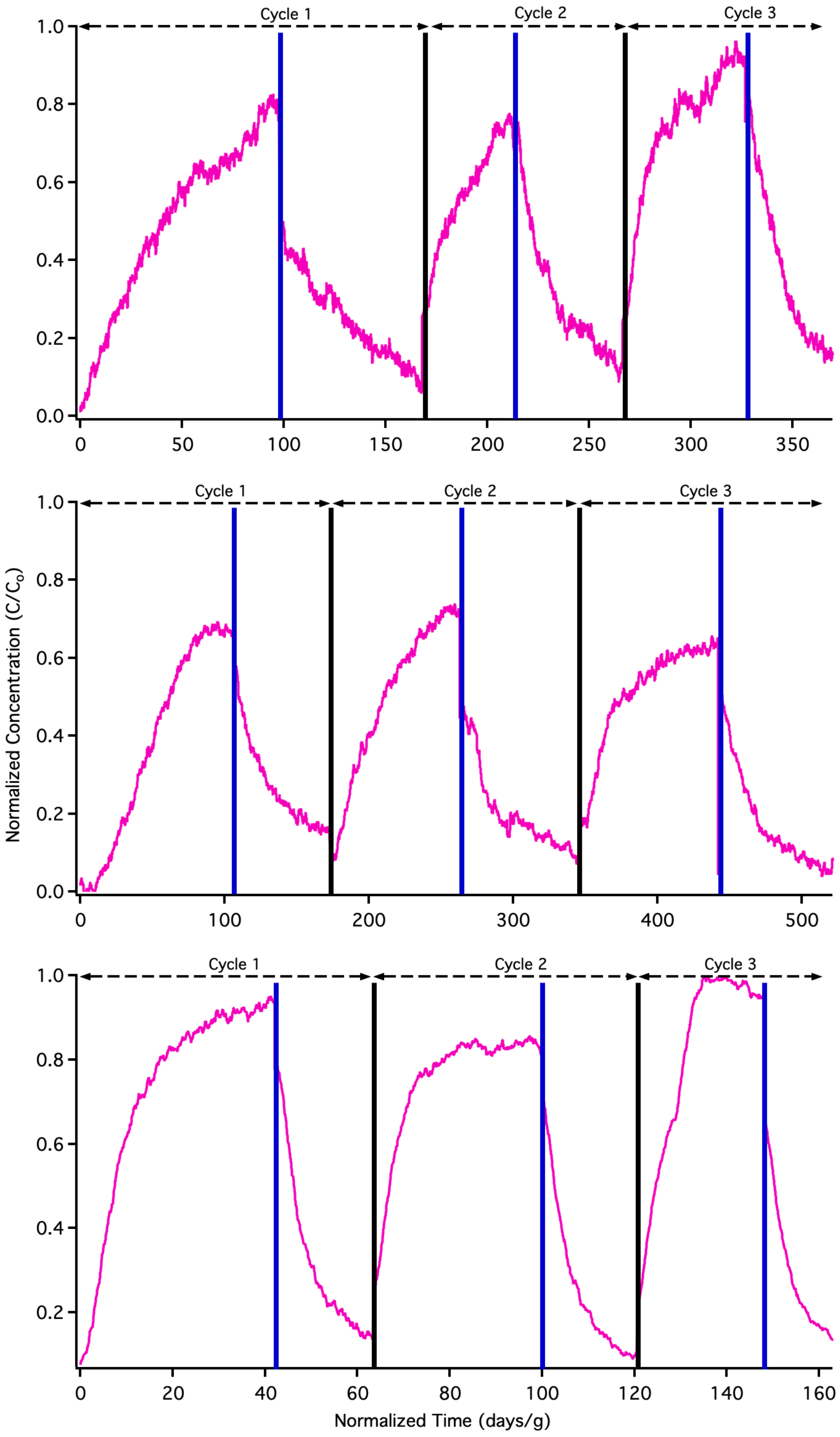

Fig. S6. Nitrous acid breakthrough curves for three samples of $Zn_2(BDC\text{-}NH_2)_2(DABCO)$. Each sample was run through 3 sorption and desorption cycles under 50% RH. Blue lines separate sorption and desorption curves, while black lines separate different cycles on the same MOF sample.

S4.5 NMR Data for Pre/Post-Nitrous Acid Exposure

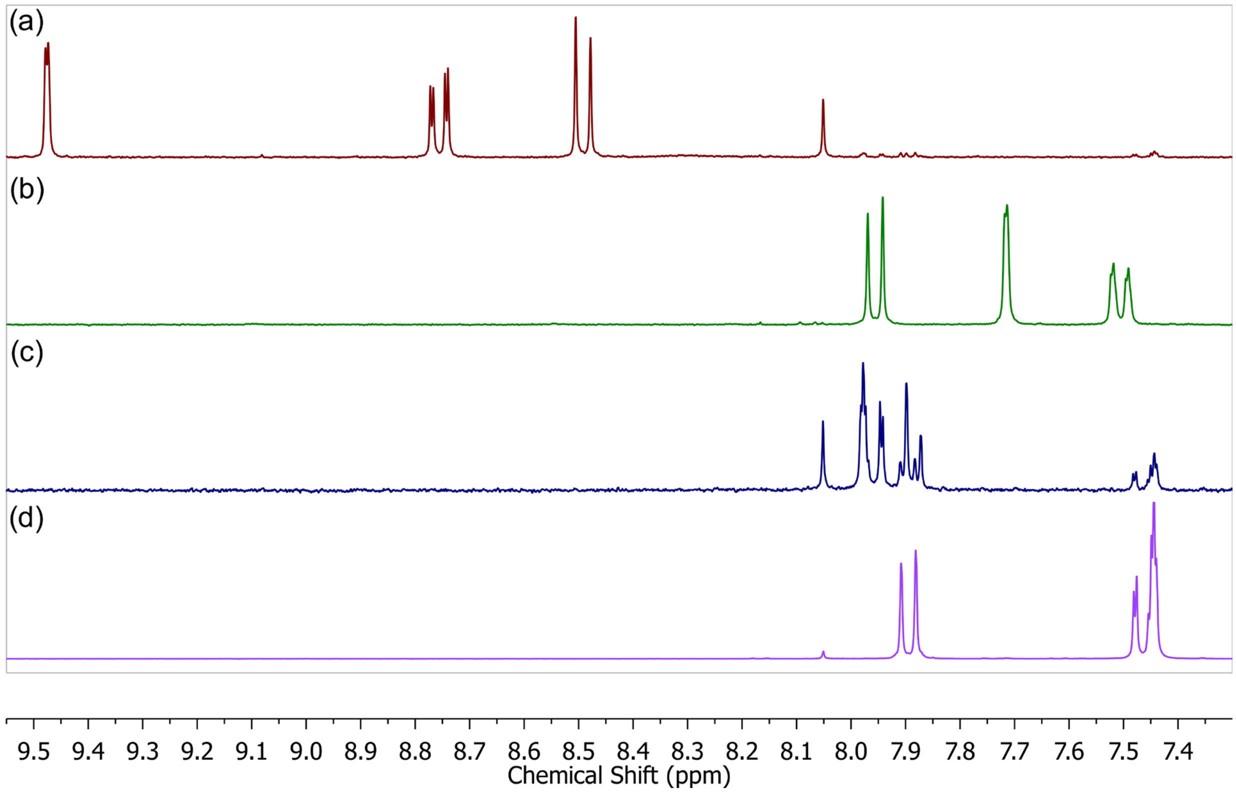

Fig. S7: High concentration (> 1 ppmv) nitrous acid experiment analyzed via solution-phase NMR. (a) UiO-66-NH_2 post nitrous acid exposure showing the formation of only UiO-66-$(N_2^+)^-$ (b) UiO-66-NH_2 prior to nitrous acid exposure. (c) UiO-66-$(N_2^+)^-$ after air exposure for 3 weeks illustrating that the diazonium salt has been converted to new products (likely UiO-66-OH as well as UiO-66-Cl). (d) H_2BDC-OH.

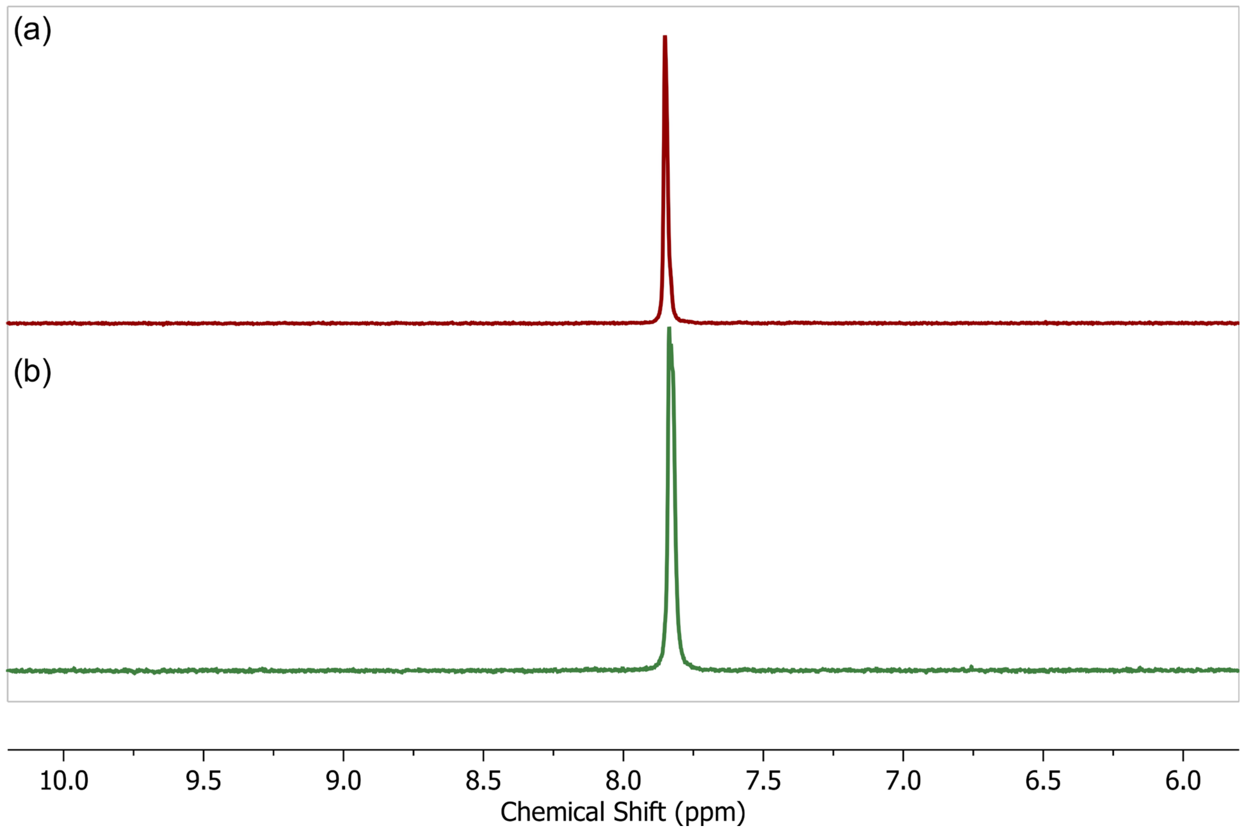

Fig. S8: Solution phase NMR measured in DMSO-d_6 (lock solvent) with 2 drops of D_2SO_4 of (a) UiO-66 pre-nitrous acid exposure and (b) UiO-66 post-nitrous acid exposure. Note that there are no additional peaks in the NMR to suggest a chemical transformation of the BDC linker.

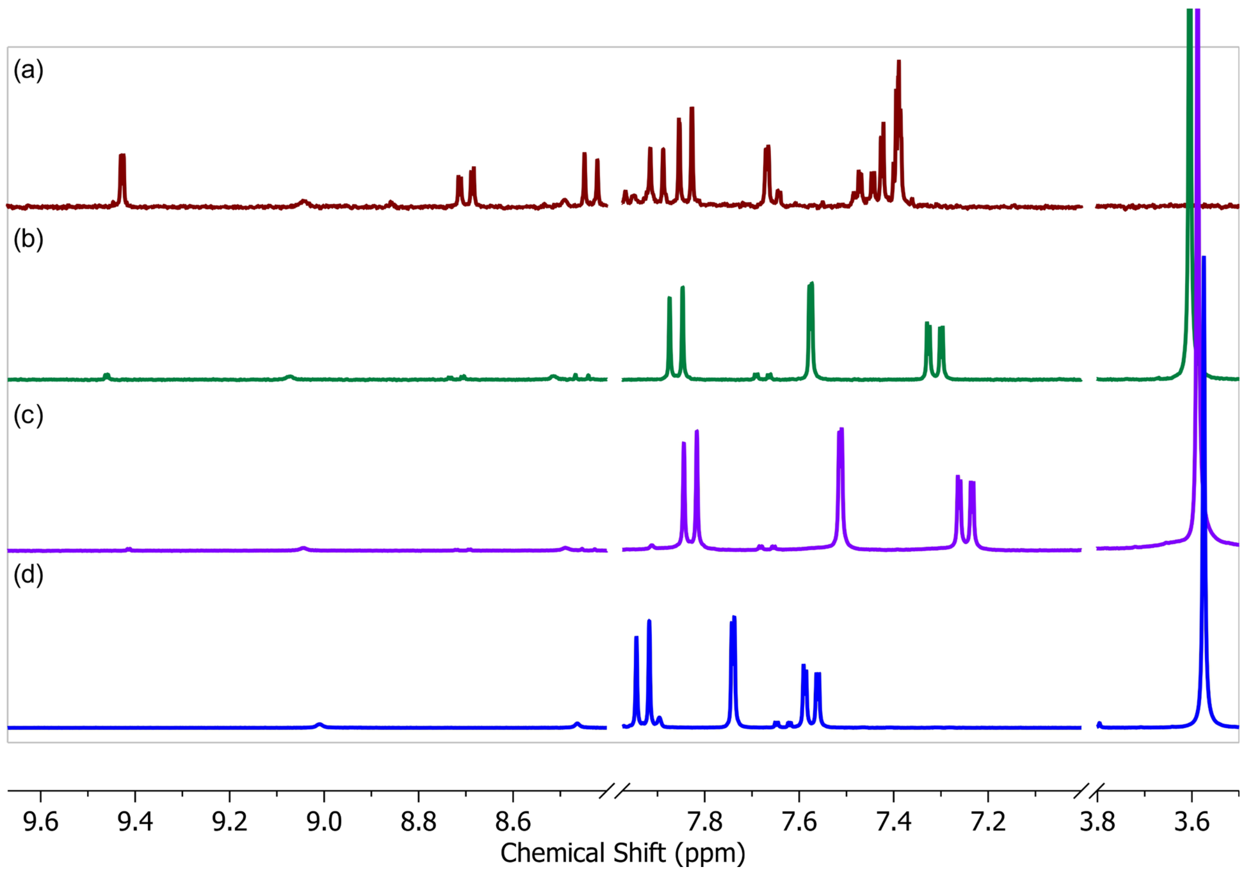

Fig. S9: Solution phase NMR measured in in DMSO-d_6 (lock solvent) with 2 drops of D_2SO_4 of (a) UiO-66-NH_2 (same as Fig. 2a in the main text) for comparison. (b)/(c) Zn_2(BDC-NH_2)$_2$DABCO measured after different concentration of nitrous acid exposure. (d) Zn_2(BDC-NH_2)$_2$DABCO measured before nitrous acid exposure. The change in chemical shift between Zn_2(BDC-NH_2)$_2$DABCO samples is due to changes in the solvent due to the effects of D_2SO_4 concentration on the linker; DABCO and BDC-NH_2 are confirmed via the addition of DABCO and H_2BDC-NH_2. Note that for the post-exposed Zn_2(BDC-NH_2)$_2$DABCO samples, there is no evidence of BDC-OH suggesting that BDC-NH_2 did not react with HONO in Zn_2(BDC-NH_2)$_2$DABCO. Furthermore, the small amount of BDC-N_2^+ that may be present, is likely due to homogeneous reaction between BDC-NH_2 and adsorbed nitrous acid during D_2SO_4 dissolution; this also confirms that the chemistry observed in the main text is heterogeneous in origin.

S4.6 IR Data for Pre/Post-Nitrous Acid Exposure

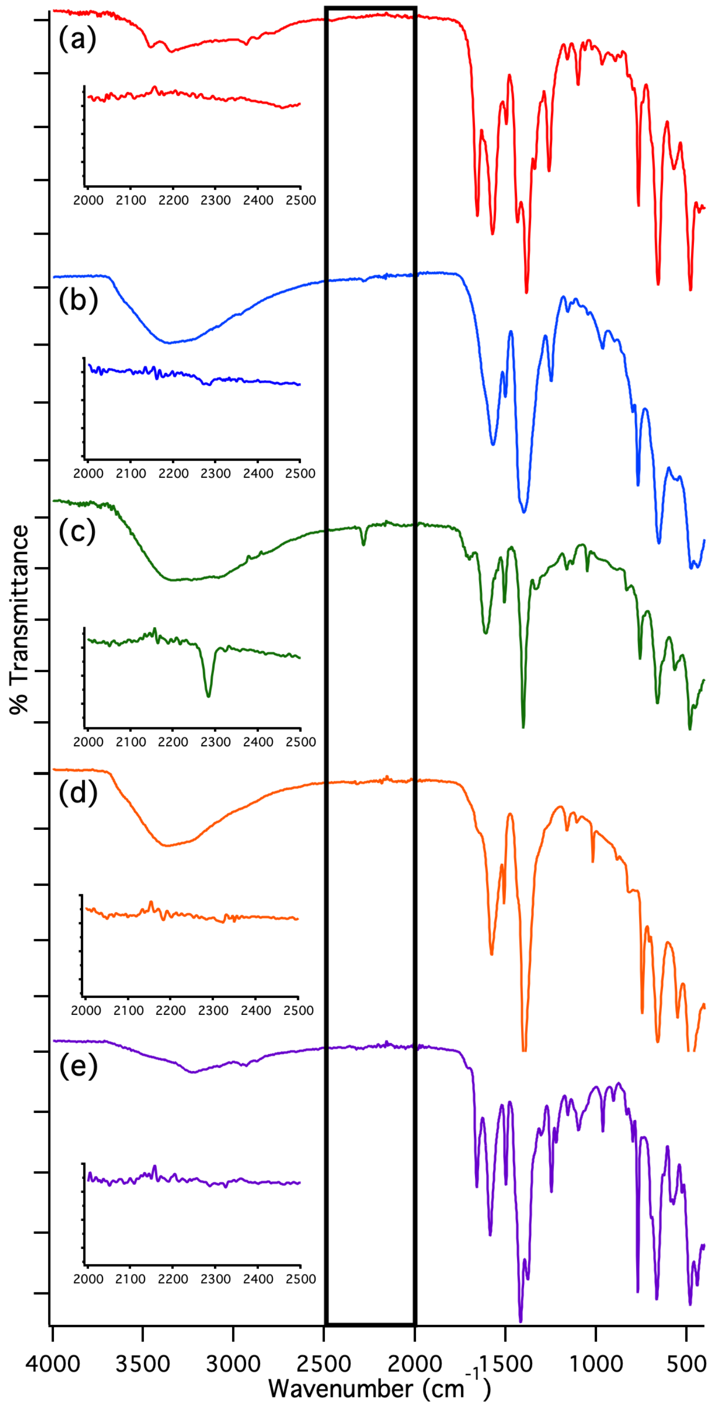

Fig. S10: (a) IR of UiO-66-NH_2. (b) IR of UiO-66-NH_2 post-moderate concentration (ca. 10x larger than low concentration exposures; ranging between 650 and 850 ppbv) nitrous acid exposures. (c) IR of UiO-66-NH_2 post-high concentration (> 1 ppmv) nitrous acid exposures. (d) IR of UiO-66 post-low (ranging from 35 to 85 ppbv) concentration nitrous acid exposures. (e) IR of UiO-66-OH. Note the presence of new vibration at 2283 cm^{-1} in both blue and green traces that is consistent with the formation of a diazonium salt.

S4.7 Gas Adsorption Data for Pre/Post-Nitrous Acid Exposure

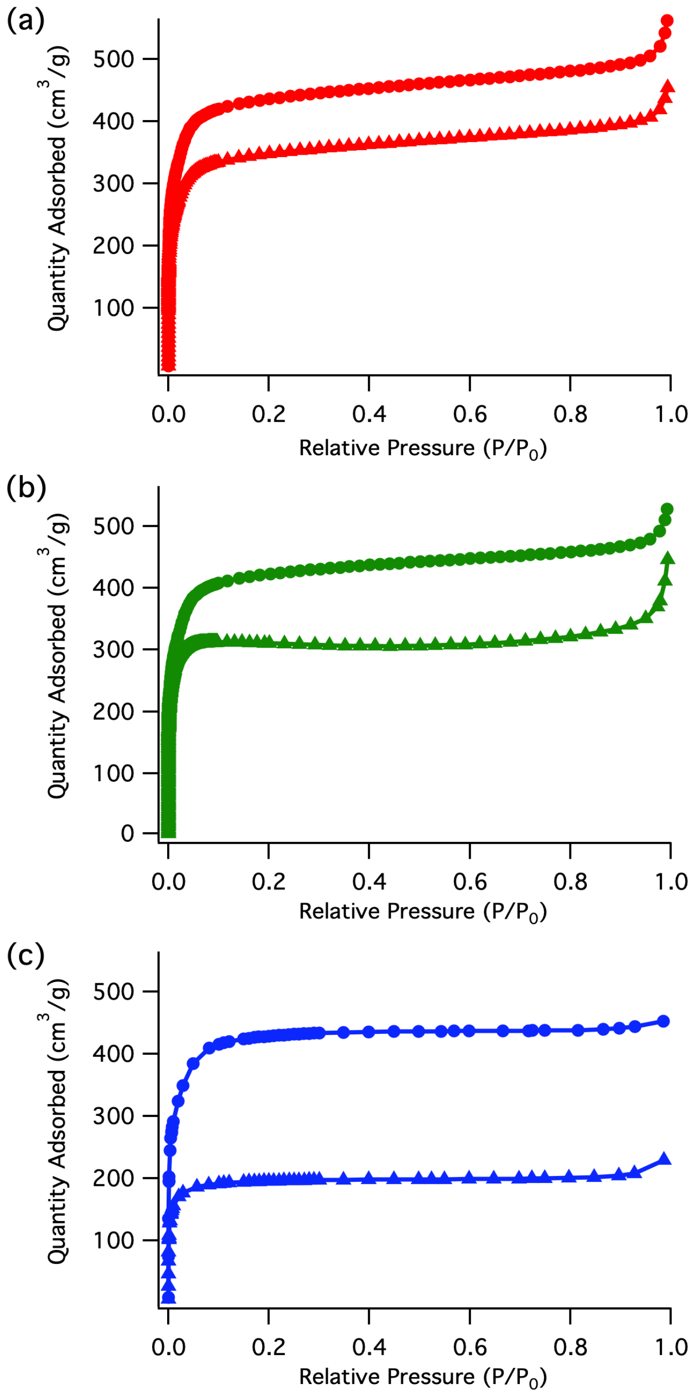

Fig. S11: N_2 gas adsorption isotherms measured at 77 K for (a) UiO-66 pre-HONO exposure (red circles), UiO-66 post-moderate (650 and 850 ppbv) HONO exposure (red triangles), (b) UiO-66-$(NH_2)_{1/6}$ pre-HONO exposure (green circles), UiO-66-$(NH_2)_{1/6}$ post-moderate HONO exposure (green triangles), (c) UiO-66-NH_2 pre-HONO exposure (blue circles), and UiO-66-NH_2 post-high (> 1 ppmv) HONO exposure (blue triangles).

S4.8 PXRD Data Pre/Post-Nitrous Acid Exposure

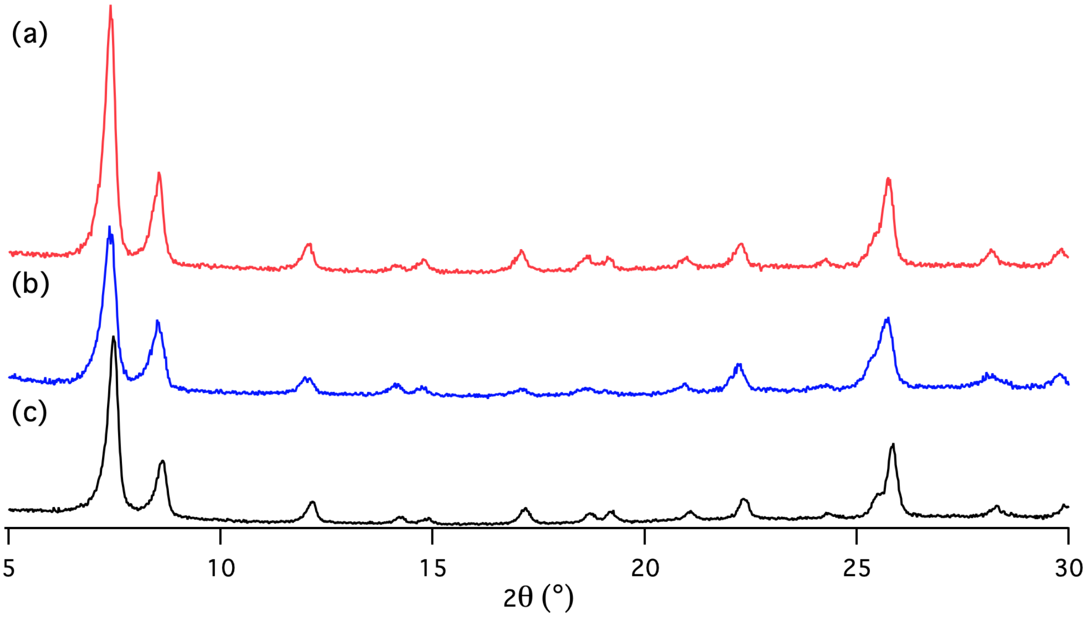

Fig. S12: (a) PXRD of UiO-66 post-high concentration (> 1 ppmv) HONO exposure. (b) PXRD of UiO-66-NH_2 post-high concentration HONO exposure. (c) PXRD for UiO-66.

S5 References

1. Y. Zhao, H. Wu, T. J. Emge, Q. Gong, N. Nijem, Y. J. Chabal, L. Kong, D. C. Langreth, H. Liu, H. Zeng and J. Li, *Chem. Eur. J.*, 2011, **17**, 5101-5109.
2. M. J. Katz, Z. J. Brown, Y. J. Colon, P. W. Siu, K. A. Scheidt, R. Q. Snurr, J. T. Hupp and O. K. Farha, *Chem. Commun.*, 2013, **49**, 9449-9451.
3. K. M. Zwolinski, P. Nowak and M. J. Chmielewski, *Chem. Commun.*, 2015, **51**, 10030-10033.
4. D. T. McGrath, V. A. Downing and M. J. Katz, *CrystEngComm*, 2018, **20**, 6082-6087.
5. D. A. Gomez-Gualdron, P. Z. Moghadam, J. T. Hupp, O. K. Farha and R. Q. Snurr, *J. Am. Chem. Soc.*, 2016, **138**, 215-224.
6. J. J. MacInnis, T. C. VandenBoer and C. J. Young, *Analyst*, 2016, **141**, 3765-3775.
7. A. Febo, C. Perrino, M. Gherardi and R. Sparapani, *Environ. Sci. Technol.*, 1995, **29**, 2390-2395.
8. Z. Hu, B. J. Deibert and J. Li, *Chem. Soc. Rev.*, 2014, **43**, 5815-5840.
9. J. B. DeCoste and G. W. Peterson, *Chem. Rev.*, 2014, **114**, 5695-5727.
10. C. Han, W. Yang, Q. Wu, H. Yang and X. Xue, *Environ. Sci. Technol.*, 2016, **50**, 5017-5023.
11. T. C. VandenBoer, S. S. Brown, J. G. Murphy, W. C. Keene, C. J. Young, A. A. P. Pszenny, S. Kim, C. Warneke, J. A. de Gouw, J. R. Maben, N. L. Wagner, T. P. Riedel, J. A. Thornton, D. E. Wolfe, W. P. Dubé, F. Öztürk, C. A. Brock, N. Grossberg, B. Lefer, B. Lerner, A. M. Middlebrook and J. M. Roberts, *J. Geophys. Res. Atmos.*, 2013, **118**, 10155-10171.
12. T. C. VandenBoer, C. J. Young, R. K. Talukdar, M. Z. Markovic, S. S. Brown, J. M. Roberts and J. G. Murphy, *Nat. Geosci.*, 2015, **8**, 55-60.
13. B. K. Place, C. J. Young, S. E. Ziegler, K. A. Edwards, L. Salehpoor and T. C. Vandenboer, *Atmos. Environ.*, 2018, **191**, 360-369.
